# 

**Published:** 1841-04

**Authors:** 


### CONTENTS OF No. XXII.

OF THE

### British and Foreign Medical Review.

APRIL, 1841.

### Part First.—Analytical and Critical Reviews.

PAGE

Art. I.— G. Valentin de Functionibus Nervorum Cerebralium et Nervi Sympathici . . . . . . . 277
On the Functions of the Cerebral Nerves and the Sympathetic. By G. Valentin.

Art. II.—Traité de Pathologie externe et de Médecine opératoire. Par Aug. Vidal . . . . . . . . 305
Treatise on External Pathology and Operative Medicine. By Aug. Vidal.
2. Handbuch der Chirurgie zum Gebrauche bei seinen Vorlesungen. Von Maximilian Joseph Chelius . . . . . . ib.
Manual of Surgery for use at his Lectures. By M. J. Chelius.
3. Elements of Surgery. By Robert Liston . . . . . ib.
4. A Dictionary of Practical Surgery. By Samuel Cooper . . . ib.

Art. III.—On the Nature and Treatment of Stomach and Urinary Diseases; being an Inquiry into the Connexion of Diabetes, Calculus, and other affections of the Kidney and Bladder with Indigestion. By William Prout, m.d.f.r.s. &c. . . . . . . . 330

Art. IV.—1. Statistical Reports on the Sickness, Mortality, and Invaliding among the Troops in Western Africa, St. Helena, the Cape of Good Hope, and the Mauritius; prepared from the records of the Army Medical Department and War-office Returns. Presented to both Houses of Parliament by Command of her Majesty . . . . . . . . 364
2. Traité des Maladies des Européens dans les Pays chauds, et spécialement au Sénégal; ou Essai Statistique Médical et Hygiènique sur le Sol, le Climat, et les Maladies de cette partie de l'Afrique. Par J. P. F. Thevenot . ib.
Treatise on the Diseases of Europeans in Warm Countries, and especially in Senegal; or a Statistical, Medical, and Hygienic Essay on the Soil, the Climate, and the Diseases of that part of Africa. By J. P. F. Thevenot.
3. Practical Medico-Historical Account of the Western Coast of Africa; embracing a Topographical Description of its Shores, Rivers, and Settlements, with the Causes, Symptoms, and Treatment of the Fevers of Western Africa. A similar Account respecting the other Diseases which prevail there. By James Boyle, m.c.s.l. . . . . . . . ib.

Art. V.—Medical and Physiological Commentaries. By Martyn Paine, m.d., a.m. 382

Art. VI.—Lectures on the Morbid Anatomy of the Serous and Mucous Membranes. By Thomas Hodgkin, m.d., &c. Vol. II. Part I. On the Mucous Membranes . . . . . . . 401

Art. VII.—Second Annual Report of the Registrar-General of Births, Deaths, and Marriages in England. Presented to both Houses of Parliament, by command of her Majesty . . . . . 422

§

PAGE

ART. VIII.—Organic Chemistry in its application to Agriculture and Physiology. By JUSTUS LIEBIG. Edited from the MSS. of the author by LYON PLAYFAIR. 436

Traité de Chémie organique. Par M. JUSTUS LIEBIG. Introduction. . ib.

ART. IX.—Medico-Chirurgical Transactions, published by the Royal Medical and Chirurgical Society of London. Vol. XXIII. . . . . . 447

ART. X.—Beobachtungen und Bemerkungen über Gehirnerweichung. Von Dr. C. H. FUCHS . . . . . . . 464

Observations and Remarks upon Softening of the Brain. By Dr. C. H. FUCHS.

ART. XI.—1. De Strabismo Dissertatio, quam scripsit et pro licentia summos in arte medica honores postea rite capessendi publico eruditorum examini modeste submittit NATH. GERS. MELCHIOR . . . . 474
Dissertation on Strabismus, &c. By NATH. GERS. MELCHIOR.
2. A Practical Treatise on the Cure of Strabismus, or Squint, by Operation, and by milder Treatment; with some new views of the Anatomy and Physiology of the Muscles of the Human Eye. By P. BENNETT LUCAS . ib.
3. Practical Remarks on the New Operation for the Cure of Strabismus, or Squinting. Illustrated with lithographic Engravings. By EDWARD W. DUFFIN . . . . . . . ib.
4. Die Behandlung des Schielens durch den Muskelschnitt. Ein Sendschreiben an Herrn Geheimrath Professor Ritter Dr. Dieffenbach zu Berlin. Von Dr. F. A. v. AMMON . . . . . . . ib.
The Treatment of Strabismus by Myotomy. An Epistle to Professor Dieffenbach, &c., of Berlin. By Dr. F. A. Von AMMON.
5. Practical Hints on the cure of Squinting by Operation. By F. W. GRANT CALDER . . . . . . . . ib.
6. Du Strabisme. Par le Docteur CHARLES PHILLIPS . . . ib.
On Strabismus. By Dr. CHARLES PHILLIPS.
7. Division of both Internal Recti in Squinting, in preference to that of any other Muscle, when the Division of one Adductor only is not instantly and completely successful. By THOMAS ELLIOT, M.R.C.S.L. . . . ib.
Division of the Corresponding Recti Muscles of both Eyes in Strabismus. By THOMAS ELLIOT, M.R.C.S.L.
8. Some Remarks on Strabismus, including an Analysis of Two Hundred Cases. By C. RADCLYFFE HALL . . . . . . . ib.

ART. XII.—The Anatomy of the Nerves of the Uterus. By ROBERT LEE, M.D.F'R.S. . . . . . . . . 493

ART. XIII.—Human Physiology. By JOHN ELLIOTSON, M.D., F.R.S., &c. &c. With which is incorporated much of the elementary part of the Institutiones Physiologicæ of J. F. Blumenbach, M.D., F.R.S. . . . 497

ART. XIV.—Commentatio de Tumoribus in Pelvi, Partum impedientibus, a gratioso medicorum ordine Heidelbergensi præmio ornata. Auctore B. R. PUCHELT, Med.-Chir. et Obstetr. Doctore, cum præfatione F. C. NAEGELE. . 500
A Treatise on Tumours in the Pelvis as a cause of difficult labour; being the essay to which the prize was adjudged by the medical faculty of the University of Heidelberg. By Dr. B. R. PUCHELT. With a preface by F. C. NAEGELE, M.D. &c. &c.

### PART SECOND.—Bibliographical Notices.

ART. I.—A Discourse on the Phenomena of Sensation, as connected with the Mental, Physical, and Instinctive Faculties of Man. By JAMES JOHNSTONE, M.D. . . . . . . . . . 505

ART. II.—Treatise on the Sympathetic Relation between the Stomach and the Brain, and, throughout, between the Digestive and the Nervous Systems, in the Causation and Cure of Diseases. With an Appendix containing a few Observations on certain points connected with the Treatment of Chronic Disease, and its attendant Debility. By CHARLES WIGHTMAN, M.D. . 506

PAGE

ART. III.—Elements of Physiology, for the use of Students, and with especial reference to the wants of Practitioners. By RUDOLPH WAGNER, M.D. Translated from the German, with additions, by ROBERT WILLIS, M.D. Part I. On Generation and Development . . . . . 507

ART. IV.—A Practical Treatise on the Venereal Disease; founded on Six Lectures on that subject, delivered in the Session of 1838-9, at the Aldersgate-street School of Medicine. By F. C. SKEY, F.R.S. . . . 508

ART. V.—An Enquiry into the Efficacy of Digitalis in the treatment of Idiopathic Epilepsy. By EDMOND SHARKEY, A.B. M.D. . . . . 509

ART. VI.—Elements of Electro-Metallurgy, or the Art of working in metals by the Galvanic fluid, &c. By ALFRED SMEE . . . . . 510

ART. VII.—The Domestic Management of the Sick Room, necessary in aid of Medical Treatment for the Cure of Diseases. By ANTHONY TODD THOMSON, M.D. F.L.S. &C. . , . . . . . 511

ART. VIII.—Essay on Tobacco. By HENRY WILSON CLELAND, M.D. . . 512

ART. IX.—Descriptive Catalogue of the Preparations in the Museum of the Royal College of Surgeons in Ireland. By JOHN HOUSTON, M.D., M.R.I.A., Vol. II. (PATHOLOGY). . . . . . . 513

ART. X.—A Lecture on Loxatrbus or Club-foot. By T. D. MÜTTER, M.D. . ib.

ART. XI.—Elements of Chemistry, including the most recent discoveries and applications of the Science to Medicine and Pharmacy, and to the Arts. By ROBERT KANE, M.D., M.R.I.A. &C. . . . . . . 514

ART. XII.—Observations on the Management of Madhouses. Part II. Containing an Account of Susannah Roginson, &c. By CALEB CROWTHER, M.D. . 515

ART. XIII.—Historisch-pathologische Untersuchungen, als beiträge zur geschichte der Volskrankheiten. Von Dr. H. HAESER . . . . . ib.
Historico-pathological Researches, being contributions to the History of Epidemics. By Dr. H. HAESER.

ART. XIV.—Leçons de Clinique Médicale faites à l'Hôtel Dieu de Paris. Par M. le Professeur CHOMEL, recueiliées et publiées par F. SESTIER, M.D. Tome troisième. (PNEUMONIE) . . . . . . 516
Lectures on Clinical Medicine. By Professor CHOMEL, reported and published by F. SESTIER, M.D. &C. Third Volume. (PNEUMONIA.)

---

### PART THIRD.—Selections from the British and Foreign Journals.

### I. THE FOREIGN JOURNALS.

#### ANATOMY AND PHYSIOLOGY.

Professor ARNOLD'S Observations and Experiments on the Functions of the Pneumogastric and Spinal Accessory Nerves . . . . . 517
M. LALLEMAND on the Origin and Mode of Development of Zoospermata . 520
Dr. ADOLPH HANNOVER on the Use of Chromic Acid in Microscopic Investigations ib.
S. PAPPENHEIM on Ciliary Motion . . . . . . . . 521
A. W. VOLKMANN on the Action of the Oblique Muscles of the Eye . . ib.
Dr. MARSHALL HALL on the Vis Nervosa of Haller . . . . . 522

#### PATHOLOGY, PRACTICAL MEDICINE, AND THERAPEUTICS.

M. FERD. HOEFER'S Observations on the Employment of Platina in Medicine . 523
M. JAMES on complete Paralysis of the fifth pair on one side, with complete Abolition of Sight, Hearing, Smelling, and Taste on the same side, cured by Galvanism . . . . . . . . . 524

PAGE

M. Delmas on the Employment of the Sulphate of Alumina in the Ulcerations and Inflammations of Mucous Membranes . . . 524
M. Barth on Fibres in the walls of the Gall-Bladder . . . . ib.
Dr. Michéa on Acephalocysts in the Brain . . . . ib.

SURGERY.

M. Begin on Partial Injury of one of the Halves of the Spinal Marrow . . 525
Dr. Goyrand on the Removal of Foreign Bodies in the Joints . . 526
M. Saussiers successful performance of Tracheotomy in a case of Croup . . ib.
M. Gaudriot on the Efficacy of Chloride of Zinc in the Treatment of Gonorrhœa 527
M. Jubert on Ligature of the common Carotid Artery, &c. . . . 528
Dr. Wiesner on Stone in the Tonsils . . . . . ib.
Dr. Lehmann on Radical Cure of Varicocele by means of Invagination and Shortening of the Scrotum . . . . . . . 529
M. Ricord on the Mode of Action of Cubebs and Copaiba . . . ib.
Dr. Forget's Practical Considerations on the Treatment of White Swellings . ib.
M. Bonnet's Extraction of a Prune-stone, which had remained eleven days in the Air-Passages . . . . . . . 530

MIDWIFERY.

Dr. Königsfeld's Cæsarean Operation saving the Lives of both Mother and Child ib.
Professor von D'Outrepont's Singular Case of Tumour in the Pelvis . 531

MEDICAL POLICE.

Dr. Schweig on Kyanised Timber . . . . . . 532

CHEMISTRY.

F. Buehlmann on a peculiar Substance occurring on the Human Teeth . . 533

II. THE BRITISH JOURNALS.

The Edinburgh Medical and Surgical Journal . . . . . 534
Edinburgh Monthly Journal of Medical Science . . . . ib.
The Dublin Journal of Medical Science . . . . . 535
The Lancet . . . . . . . 536
The London Medical Gazette . . . . . . 538
The Dublin Medical Press . . . . . . 539
Provincial Medical and Surgical Journal . . . . 540
The London, Edinburgh, and Dublin Philosophical Magazine . . . ib.
Dr. John Reid on the Structure of the Placenta . . . . ib.
Dr. John Reid on some Points in the Anatomy of the Medulla Oblongata . 542
Dr. W. A. F. Browne, on Bleeding in Mania . . . . 543
Dr. Golding Bird on Flexible Stethoscopes . . . . 544

Part Fourth.—Medical Intelligence.

London Mortality Tables for the Quarter ending January 2 . . . 545
On Seclusion, as employed at Hanwell in the Treatment of the Insane . 546
OBITUARY.—Sir Astley Cooper, his Life, Character, and Writings . 549
Books received for Review . . . . . . . 553
Title, Index, &c.

APPENDIX—Thackeray Prize Essay.

